# Comparing central venous access strategies in elderly patients with dysphagia: a survival analysis

**DOI:** 10.3389/fnut.2026.1765556

**Published:** 2026-04-08

**Authors:** Yuetong Rong, Tao Liu, Dan Li, Heli Zhang, Guoqing Cui, Baohua Li, Shaomei Shang

**Affiliations:** 1 Department of Rehabilitation Medicine, Peking University Third Hospital, Beijing, China; 2 Peking University School of Nursing, Beijing, China; 3 Department of Nursing, Peking University Third Hospital, Beijing, China

**Keywords:** central venous catheter, dysphagia, frailty, older adults, parenteral nutrition, survival analysis

## Abstract

**Background:**

Dysphagia in older adults often necessitates long term parenteral nutrition (TPN). However, the optimal central venous access strategy, including implantable central venous access port (ICVAP), non-tunneled central venous catheter (NT-CVC) or peripherally inserted central catheter (PICC), remains uncertain, particularly regarding survival and complication risk.

**Methods:**

This retrospective cohort study included 73 patients aged ≥65 years with dysphagia who received parenteral nutrition via ICVAP, NT-CVC, or PICC. Device selection was based on patient/family request and feasibility/acceptability at the discharge destination. Baseline characteristics were compared using ANOVA and chi-square/Fisher’s exact tests, and standardized mean differences (SMD) were reported to quantify baseline imbalance. Survival was evaluated using Kaplan–Meier curves with log-rank tests. Multivariable Cox proportional hazards models were performed to adjust for predefined covariates. Short-term mortality and complications were compared using chi-square or Fisher’s exact tests.

**Results:**

Survival differed significantly among the three groups (log-rank *p* < 0.001), with median survival of 305 days for ICVAP, 195 days for PICC, and 43 days for NT-CVC. After adjustment, NT-CVC was associated with a significantly higher mortality risk compared to ICVAP (HR = 4.15, 95% CI: 1.17–14.78, *p* = 0.028), while no significant difference was observed between PICC and ICVAP (HR = 1.05, 95% CI: 0.36–3.04, *p* = 0.927). Independent predictors of mortality included advanced age (HR = 1.08, *p* = 0.004), lower BMI (HR = 0.89, *p* = 0.034), higher CFS (HR = 3.70, *p* = 0.027), and total lymphocyte count (TLC) (HR = 1.00, *p* = 0.009). NT-CVC had the highest short-term mortality (34.6% at 30 days, 73.1% at 90 days, *p* < 0.05). No significant differences were observed in pneumonia or sepsis rates.

**Conclusion:**

In this cohort of elderly patients with dysphagia receiving long-term parenteral nutrition, ICVAP use was associated with longer survival compared with NT-CVC, while no significant survival difference was observed between PICC and ICVAP. Given the retrospective design and non-random catheter selection, these findings should be interpreted as associations and warrant confirmation in prospective, multicenter studies incorporating standardized complication definitions and patient-centered outcomes.

## 1 Introduction

Dysphagia is increasingly recognized as a geriatric syndrome, with a prevalence exceeding 48% among older adults (1). It is associated with poor outcomes, including malnutrition, dehydration, aspiration pneumonia and a one-year mortality rate over 25%, especially in frail individuals (2, 3). Total parenteral nutrition (TPN) is often necessary for patients with severe swallowing impairment or contraindications to enteral feeding (4). However, the optimal central venous access route for TPN delivery in this population remains uncertain (5, 6). Current guidelines offer limited recommendations on venous access selection for long-term parenteral nutrition in frail elderly patients with dysphagia (1).

Three commonly used devices include implantable central venous access port (ICVAP), non-tunneled central venous catheter (NT-CVC), and peripherally inserted central catheter (PICC), each with different complication profiles (7). Prior studies have mainly focused on short-term risks such as catheter-related bloodstream infections (CRBSI), but data on long-term survival are limited (8). Frailty, often under-assessed in prior studies, may act as a key modifier of survival outcomes related to catheter choice.

Survival is a critical endpoint in this population, reflecting not only catheter safety but also overall management effectiveness. This study aims to compare long-term and short-term survival, as well as complication rates, among elderly patients with dysphagia receiving TPN via ICVAP, NT-CVC, or PICC. By adjusting for key prognostic variables, this study seeks to provide clinical decision-making regarding optimal central venous access in this vulnerable population.

## 2 Materials and methods

### 2.1 Data source and ethical approval

This study was a secondary analysis based on data obtained from the Dryad Digital Repository (DDR), originally collected at Miyanomori Memorial Hospital, Japan. DDR is a publicly accessible scientific database widely recognized for multidisciplinary data sharing (9). The dataset, titled Comparison of Long-Term Outcomes Between Enteral Nutrition via Gastrostomy and Total Parenteral Nutrition in Older Persons with Dysphagia, includes clinical data from elderly patients who received nutritional support between January 2014 and January 2017 (10).

The original study was approved by the Miyanomori Memorial Hospital Ethics Committee, with informed consent waived due to anonymized data. For the current study, we extracted a subset of patients receiving TPN and reanalyzed the data to assess survival outcomes associated with different central venous access devices.

### 2.2 Participants

We included patients aged ≥65 years with clinically confirmed dysphagia who required long-term TPN at Miyanomori Memorial Hospital between January 2014 and January 2017. Dysphagia was diagnosed based on clinical evaluation and documented inability to maintain adequate oral intake. Inclusion criteria were: (1) age 65 years, (2) indication for TPN due to dysphagia, and (3) available follow-up data. Exclusion criteria were: (1) advanced malignancy, (2) PEG placement for decompression purposes only, and (3) history of PEG prior to 2014.

A total of 73 eligible patients were categorized into three groups based on venous access type: ICVAP (*n* = 28), NT-CVC (*n* = 26), and PICC (*n* = 19). In TPN cases, the choice among ICVAP, NT-CVC, and PICC was made based on the patient’s or their family’s request and the feasibility and acceptability of each catheter type at the discharge destination; nutritional prescriptions were determined by clinicians based on clinical evaluations.

### 2.3 Covariates

Baseline covariates included demographics (age, sex), nutritional status [body mass index (BMI), serum albumin (ALB), total cholesterol (TC), hemoglobin (Hb), C-reactive protein (CRP)], frailty [clinical frailty scale (CFS)], comorbidities [cerebrovascular disease (CVD), dementia, neuromuscular disease (NMD), ischemic heart disease (IHD), chronic heart failure (CHF), chronic lung disease (CLD), chronic kidney disease (CKD), aspiration pneumonia (AP)], and immune status [total lymphocyte count (TLC)]. The primary outcome was long-term survival, defined as time from catheter placement to all-cause death. Secondary outcomes included 30- and 90-day mortality, as well as major complications such as pneumonia and sepsis.

### 2.4 Statistical analysis

All statistical analyses were performed using R software (version 4.2.1). Baseline characteristics were compared across groups using analysis of variance (ANOVA) for continuous variables and chi-square or Fisher’s exact tests for categorical variables. In addition to *p-*values, standardized mean differences (SMD) were calculated to quantify the magnitude of between-group baseline imbalance, with an absolute SMD > 0.20 indicating meaningful imbalance. Kaplan–Meier survival curves were constructed to illustrate unadjusted survival distributions, and differences between groups were assessed using the log-rank test. Multivariable Cox proportional hazards models were used to estimate hazard ratios (HR) and 95% confidence intervals (CI) for mortality, adjusting for all predefined covariates. The proportional hazards assumption was tested using Schoenfeld residuals. Secondary outcomes (30-day and 90-day mortality, pneumonia, and sepsis rates) were compared using chi-square or Fisher’s exact tests. Bonferroni correction was applied to adjust for multiple comparisons where applicable. Statistical significance was defined as two-sided *p* < 0.05.

## 3 Results

### 3.1 Baseline characteristics

Among the 73 elderly patients with dysphagia receiving TPN, 28 (38.4%) received ICVAP, 26 (35.6%) received NT-CVC, and 19 (26.0%) received PICC. The mean age was 86.77 ± 6.72 years and 61.6% were female. Baseline comparability was assessed using standardized mean differences in addition to *p* values.

Meaningful baseline imbalances were observed for TLC (SMD = 0.874, *p* < 0.001), CKD (SMD = 0.662, *p* = 0.002), BMI (SMD = 0.609, *p* = 0.003), CHF (SMD = 0.597, *p* = 0.013), AP (SMD = 0.508, *p* = 0.025), IHD (SMD = 0.457, *p* = 0.038) and dementia (SMD = 0.474, *p* = 0.077). Other laboratory measures showed smaller between-group differences, including serum ALB (SMD = 0.181, *p* = 0.496), TC (SMD = 0.180, *p* = 0.541), Hb (SMD = 0.128, *p* = 0.720) and CRP (SMD = 0.286, *p* = 0.281). Detailed baseline characteristics are presented in Table 1.

**Table 1:** Baseline characteristics of elderly patients with dysphagia receiving TPN, stratified by venous access type.

Variable	Overall (*n* = 73)	ICVAP (*n* = 28)	NT-CVC (*n* = 26)	PICC (*n* = 19)	SMD	*P*-value
Age, years	86.77 (6.72)	88.57 (5.01)	85.15 (8.58)	86.32 (5.65)	0.356	0.166
Sex					0.193	0.564
Male	28 (38.4%)	9 (32.1%)	12 (46.2%)	7 (36.8%)		
Female	45 (61.6%)	19 (67.9%)	14 (53.8%)	12 (63.2%)		
BMI (kg/m^2^)	19.05 (3.67)	17.77 (2.71)	20.98 (3.89)	18.37 (3.72)	0.609	0.003*
CFS	7.88 (0.33)	7.93 (0.26)	7.88 (0.33)	7.79 (0.42)	0.267	0.369
CVD	26 (35.6%)	11 (39.3%)	11 (42.3%)	4 (21.1%)	0.312	0.297
Dementia	45 (61.6%)	18 (64.3%)	12 (46.2%)	15 (78.9%)	0.474	0.077
NMD	4 (5.5%)	1 (3.6%)	1 (3.8%)	2 (10.5%)	0.183	0.531
AP	21 (28.8%)	3 (10.7%)	11 (42.3%)	7 (36.8%)	0.508	0.025*
IHD	16 (21.9%)	4 (14.3%)	10 (38.5%)	2 (10.5%)	0.457	0.038*
CHF	37 (50.7%)	12 (42.9%)	19 (73.1%)	6 (31.6%)	0.597	0.013*
CLD	7 (9.6%)	1 (3.6%)	2 (7.7%)	4 (21.1%)	0.373	0.125
CKD	24 (32.9%)	4 (14.3%)	15 (57.7%)	5 (26.3%)	0.662	0.002*
ALB, g/dL	2.82 (0.53)	2.90 (0.42)	2.76 (0.66)	2.79 (0.50)	0.178	0.618
TLC, mm^3^	1203.11 (682.95)	1645.31 (692.99)	858.73 (443.28)	1014.87 (597.98)	0.874	<0.001*
TC, mg/dL	145.58 (43.21)	149.67 (34.97)	138.00 (51.80)	150.16 (41.73)	0.178	0.541
Hb, g/dL	10.21 (2.10)	10.37 (2.14)	9.97 (2.14)	10.30 (2.07)	0.126	0.767
CRP, mg/dL	2.92 (3.04)	2.20 (2.91)	3.31 (3.50)	3.45 (2.42)	0.286	0.281

ICVAP, implantable central venous access port; NT-CVC, non-tunneled central venous catheter; PICC, 1peripherally inserted central catheter; BMI, body mass index; CFS, clinical frailty scale; ALB, serum albumin; TLC, total lymphocyte count; TC, total cholesterol; Hb, hemoglobin; CRP, C-reactive protein; SMD, standardized mean differences. Values are presented as mean (SD) or n (%), as appropriate. *P*-values were calculated using one-way ANOVA for continuous variables or chi-square/Fisher’s exact test for categorical variables. SMD were reported to quantify baseline imbalance, with absolute SMD>0.20 indicating meaningful imbalance. Asterisks denote statistical significance at *p* < 0.05.

### 3.2 Long-term survival outcomes

**Figure 1:**
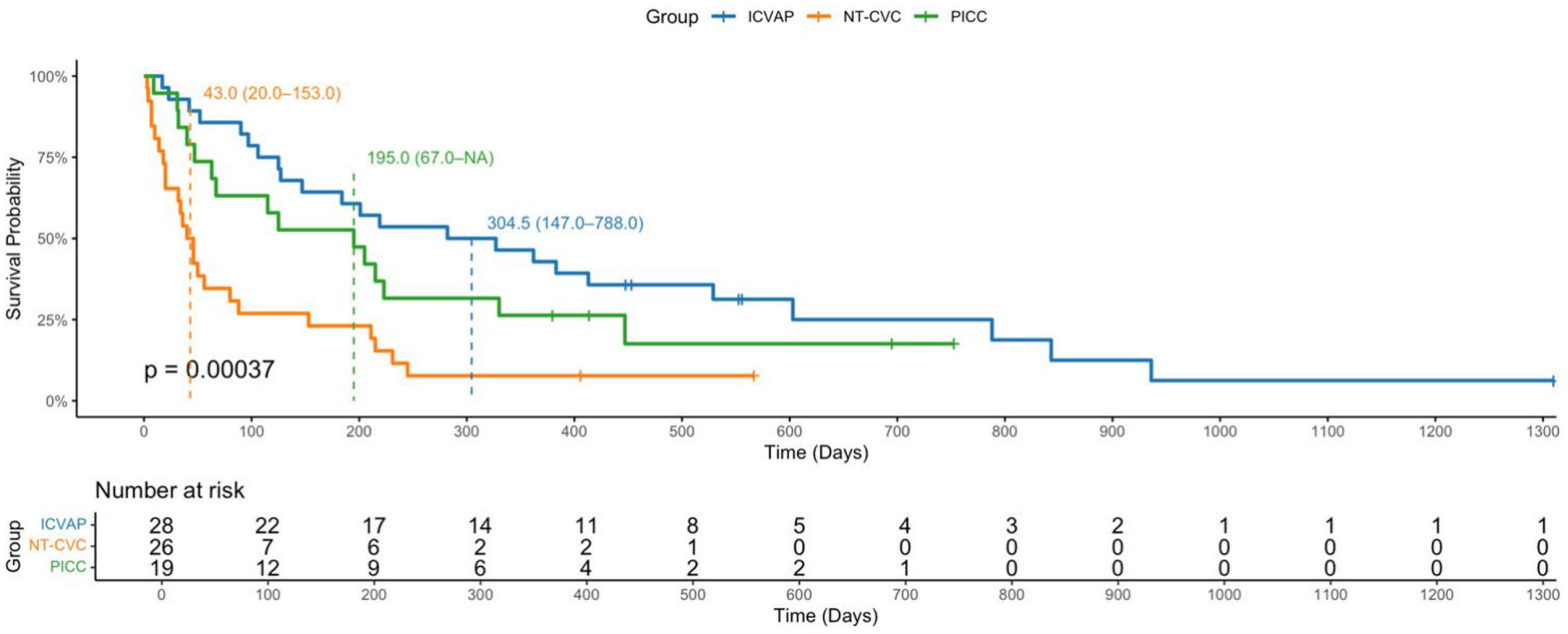
Kaplan–Meier survival curves by type of TPN in elderly patients receiving TPN. Survival curves compare long-term survival outcomes among patients with ICVAP, NT-CVC and PICC. Log-rank test *p* < 0.001.

As illustrated in Figure 1, Kaplan–Meier survival curves showed significant differences in long-term survival across the three venous access groups (log-rank <*P* 0.001). Patients with NT-CVC had the shortest median survival time (43 days, 95% CI, 20.0–153.0), followed by PICC (195 days, 95% CI, 67.0–NA), while those with ICVAP showed the longest survival (305 days, 95% CI, 147.0–788.0).

Multivariable Cox proportional hazards regression (Table 2) confirmed that NT-CVC placement was associated with a significantly higher risk of mortality compared to ICVAP (HR = 4.15, 95% CI: 1.17–14.78, *p* = 0.028). In contrast, PICC was not significantly different from ICVAP in terms of mortality risk (HR = 1.05, 95% CI: 0.36–3.04, *p* = 0.927). Among all covariates, advanced age (HR = 1.08, *p* = 0.004), lower BMI (HR = 0.89 *p* = 0.034), higher CFS (HR = 3.70, *p* = 0.027), and TLC (HR = 1.00, *p* = 0.009) were independently associated with increased mortality.

**Table 2:** Multivariable cox proportional hazards model for all-cause mortality by venous access type.

Variable	HR (95% CI)	*P*-value
NT-CVC vs. ICVAP	4.15 (1.17, 14.78)	0.028*
PICC vs. ICVAP	1.05 (0.3639, 3.0353)	0.927
Age (per year)	1.08 (1.03, 1.15)	0.004*
Sex	0.50 (0.17, 1.33)	0.164
BMI	0.89 (0.80, 0.99)	0.034*
CFS	3.70 (1.16, 11.75)	0.027*
CVD	1.39 (0.61, 3.17)	0.433
Dementia	1.43 (0.31, 3.15)	0.377
NMD	1.48 (0.31, 7.18)	0.623
AP	1.47 (0.57, 3.78)	0.423
IHD	1.19 (0.51, 2.77)	0.694
CHF	0.90 (0.45, 1.83)	0.776
CLD	1.43 (0.45, 4.58)	0.548
CKD	1.69 (0.55, 5.13)	0.358
ALB	0.58 (0.32, 1.05)	0.074
TLC	1.00 (1.00, 1.00)	0.009*
TC	1.00 (0.99, 1.01)	0.890
Hb	0.90 (0.74, 1.10)	0.316
CRP	1.06 (0.94, 1.19)	0.368

HR, hazard ratio; CI, confidence interval; other abbreviations as in Table 1. Multivariable Cox regression adjusted for all listed covariates. Asterisks indicate statistical significance at *P* < 0.05.

### 3.3 Short-term mortality and complications

As shown in Table 3, significant differences in short-term mortality were observed among the three venous access groups. The 30-day mortality rate was highest in the NT-CVC group (34.6%), followed by ICVAP (7.1%) and PICC (5.3%) (*p* = 0.010). The difference became even more pronounced at 90 days, with the NT-CVC group exhibiting a markedly higher mortality rate (73.1%) compared to the ICVAP (17.9%) and PICC (36.8%) groups (*p* < 0.001).

**Table 3:** Short-term mortality and complications during follow-up.

Short-term outcomes and complications	ICVAP (*n* = 28)	NT-CVC (*n* = 26)	PICC (*n* = 19)	*P*-value
30-day mortality, n (%)	2 (7.1%)	9 (34.6%)	1 (5.3%)	0.010*
90-day mortality, n (%)	5 (17.9%)	19 (73.1%)	7 (36.8%)	<0.001*
Pneumonia, n (%)	3 (10.7%)	7 (26.9%)	6 (31.6%)	0.140
Sepsis, n (%)	9 (32.1%)	8 (30.8%)	3 (15.8%)	0.633

Abbreviations as in Table 1. Pneumonia and sepsis were recorded as clinical diagnoses during follow-up and were not adjudicated as catheter-related infections. *p*-values were calculated using the chi-square or Fisher’s exact test. Asterisks denote statistical significance at *p* < 0.05.

In terms of complications, the incidence of pneumonia ranged from 10.7% in the ICVAP group to 31.6% in the PICC group, while sepsis occurred in 32.1, 30.8, and 15.8% of patients in the ICVAP, NT-CVC, and PICC groups, respectively. However, these differences were not statistically significant (*p* = 0.140 for pneumonia; *p* = 0.633 for sepsis).

## 4 Discussion

This study examined survival outcomes by venous access type among elderly patients with dysphagia requiring long-term TPN. In this cohort, ICVAP use was associated with longer survival compared with NT-CVC. This association remained in multivariable Cox models adjusting for clinically relevant covariates including age, frailty, nutritional indicators and comorbidities. In the PICC group, the median survival was 195 days, and survival did not differ significantly from ICVAP. These findings challenge the current tendency to default to NT-CVC in frail elderly patients and support a shift toward ICVAP as the preferred long-term access modality in this high-risk group (11). Given the retrospective design and non-random catheter selection influenced by patient’s or their family’s request and feasibility at the discharge destination, the observed differences should be interpreted as associative relationships rather than causal inferences regarding the effect of catheter type on mortality. These findings underscore that venous access selection should be clinically relevant in real-world practice for this high-risk population.

ICVAP are commonly selected when durable venous access is anticipated and when the discharge environment can support long-term catheter maintenance, aligning with guideline principles that device selection should match anticipated duration and care setting for parenteral nutrition (12, 13). From a clinical perspective, implantable ports reduce external components and may decrease opportunities for line manipulation (14, 15); however, comparative infectious risk across devices is not uniform and depends on patient populations, catheter-care protocols, and endpoint definitions (16). Therefore, while an infection-related mechanism is plausible, the survival advantage observed with ICVAP in our cohort should be interpreted cautiously and may also reflect patient selection and care-environment differences that influence both device choice and outcomes.

NT-CVC are often used when rapid access is required or when clinical status and discharge constraints limit the feasibility of long-term devices, including in emergency or intensive care unit (ICU) settings where patients have acute hemodynamic instability, potentially introducing confounding by indication (17, 18). Guideline documents for parenteral nutrition access emphasize that non-tunneled catheters are typically short-term solutions and are not preferred for prolonged or home parenteral nutrition because of higher susceptibility to complications (13, 19). The baseline imbalance observed in our cohort further complicates interpretation. For example, the NT-CVC group had a higher prevalence of chronic heart failure and lower total cholesterol, and prior evidence in advanced heart failure has reported that lower total cholesterol is associated with higher mortality risk (often described as a “cholesterol paradox”) (20). Accordingly, the markedly higher early mortality observed in the NT-CVC group may reflect a combination of baseline risk, clinical urgency, and care-setting constraints rather than a catheter-attributable causal effect (7).

PICC placement is less invasive and can be performed at the bedside, making it a pragmatic option when ICVAP is not feasible due to procedural considerations or patient factors (7, 21). For home parenteral nutrition, ESPEN practical guidance notes that PICC can be used when the expected duration is limited (e.g., estimated <6 months), whereas longer courses generally favor long-term devices (19). In our cohort, the median survival in the PICC group was 195 days, and survival did not differ significantly between PICC and ICVAP. Because patient preferences, quality of life during survival, functional trajectories, and caregiver burden were not captured in the original dataset, we could not evaluate patient-centered trade-offs that are often central to choosing between PICC and implantable devices in frail older adults.

For the safety profile of different venous access strategies, we summarized pneumonia and sepsis as clinical outcomes during follow-up. Evidence syntheses in home parenteral nutrition suggest that PICC may have lower catheter-related bloodstream infection rates than ports, while differences versus tunneled catheters are less certain, highlighting heterogeneity across devices and settings (16, 22). In our dataset, the proportion of sepsis was numerically highest in the ICVAP group, but between-group differences were not statistically significant. Importantly, pneumonia and sepsis were recorded as clinical diagnoses during follow-up and were not adjudicated as catheter-related infections; microbiological confirmation, catheter dwell time, catheter-care protocols, and temporal linkage between catheter use and infection were unavailable. Therefore, these data cannot be used to attribute sepsis risk directly to catheter type or to infer catheter-related bloodstream infection risk, and the complication findings should be interpreted as descriptive and hypothesis-generating.

## 5 Limitation

Several limitations should be acknowledged. (1) This study is a secondary analysis of a retrospective, single-center dataset with a modest and uneven sample size across groups, which may limit generalizability and the precision of effect estimates. (2) Venous access type was not randomly assigned and was influenced by patient/family request and feasibility at the discharge destination; although baseline imbalance was quantified using standardized mean differences and multivariable adjustment was performed, residual confounding cannot be excluded. (3) In the original dataset, complication assessment was limited to pneumonia and sepsis documented as clinical diagnoses during follow-up; these events were not adjudicated as catheter-related infections. (4) Broader patient-centered outcomes, including patient preferences, quality of life, functional trajectories, and caregiver burden, were not captured and therefore could not be analyzed.

## 6 Conclusion

This study suggests that, among elderly patients with dysphagia receiving long-term parenteral nutrition, ICVAP use was associated with longer survival compared with NT-CVC, while survival with PICC appeared comparable to ICVAP in this cohort. Given the retrospective, non-randomized design and baseline imbalances, these results should be interpreted as associations rather than causal effects. In practice, ICVAP may be considered when feasible, and PICC may represent an alternative option when ICVAP is not suitable. Future prospective, multicenter studies incorporating standardized complication definitions and patient-centered outcomes are needed to confirm these associations and refine venous access selection in geriatric care.

## Data Availability

The original contributions presented in the study are included in the article/supplementary material, further inquiries can be directed to the corresponding authors.
