# Correction: Comparing central venous access strategies in elderly patients with dysphagia: a survival analysis

**DOI:** 10.3389/fnut.2026.1852650

**Published:** 2026-04-24

**Authors:** Yuetong Rong, Tao Liu, Dan Li, Heli Zhang, Guoqing Cui, Baohua Li, Shaomei Shang

**Affiliations:** 1 Department of Rehabilitation Medicine, Peking University Third Hospital, Beijing, China; 2 Peking University School of Nursing, Beijing, China; 3 Department of Nursing, Peking University Third Hospital, Beijing, China

**Keywords:** central venous catheter, dysphagia, frailty, older adults, parenteral nutrition, survival analysis

Author Guoqing Cui was erroneously assigned to affiliation 2. Peking University School of Nursing, Beijing, China. This affiliation has now been removed for author Guoqing Cui and corrected to affiliation 1. Department of Rehabilitation Medicine, Peking University Third Hospital, Beijing, China

The original version of this article has been updated.

